# A PD1‐CD137L Fusion Protein More Potently Enhances Anti‐Tumour Immune Responses Than Targeting PD‐L1 and CD137 Separately

**DOI:** 10.1111/imm.70189

**Published:** 2026-08-10

**Authors:** Runze Xia, Emily Pauline Nickles, Yu Mei, Thamizhanban Manoharan, Yi Tian Png, Beijia Liu, Rui Sun, Kang Yi Lee, Gloryn Chia, Chwee Ming Lim, Herbert Schwarz

**Affiliations:** ^1^ Department of Physiology Yong Loo Lin School of Medicine, National University of Singapore Singapore Singapore; ^2^ NUS Immunology Programme Life Sciences Institute, National University of Singapore Singapore Singapore; ^3^ NUSMED Immunology Translational Research Programme National University of Singapore Singapore Singapore; ^4^ Integrative Sciences and Engineering Programme National University of Singapore Singapore Singapore; ^5^ Department of Microbiology and Immunology Yong Loo Lin School of Medicine, National University of Singapore Singapore Singapore; ^6^ Department of Pharmacy, Faculty of Science National University of Singapore Singapore Singapore; ^7^ Department of Otorhinolaryngology‐Head and Neck Surgery Singapore General Hospital Singapore Singapore

**Keywords:** CD137 ligand, costimulation, immune evasion, immunotherapy, PD‐1, tumour microenvironment

## Abstract

Tumour immune evasion frequently involves PD‐L1‐mediated inhibition of T cell activity, limiting the efficacy of PD1/PD‐L1 blockade, particularly in PD‐L1‐low or immunologically ‘cold’ tumours. To overcome this limitation, we developed a dual targeting human PD1‐CD137L (hPD1‐CD137L) fusion protein that blocks PD‐L1–PD1 inhibitory signalling and simultaneously delivers CD137‐mediated T cell costimulation. The fusion protein was produced with high purity and demonstrated specific binding to PD‐L1‐expressing tumour cells and CD137‐positive T cells. The hPD1‐CD137L retained a stable multimeric structure under acidic conditions and during prolonged storage, supporting its suitability for systemic administration and activity within the tumour microenvironment. Functionally, hPD1‐CD137L enhanced T cell activation, as evidenced by increased NF‐κB signalling and activation marker expression, and promoted robust tumour cell cytolysis in both 2D and 3D co‐culture systems. Anti‐tumour activity was observed across multiple tumour models with varying PD‐L1 expression, including nasopharyngeal carcinoma (NPC), rhabdomyosarcoma, lung carcinoma and patient‐derived colorectal cancer organoids. Notably, hPD1‐CD137L enhanced cytolysis of C666 NPC cells by patient‐derived tumour‐infiltrating lymphocytes at low effector‐to‐target ratios, and was more potent than a combination of an anti‐PD‐1 antibody and a CD137 agonist. In vivo, PBMC‐humanised NSG mice tolerated hPD1‐CD137L without significant weight loss, systemic inflammation or survival impact. Subsequent efficacy study in PBMC‐humanised, Rd18 rhabdomyosarcoma‐engrafted or C666 nasopharyngeal carcinoma‐engrafted NSG mice showed tumour growth suppression without overt toxicity. This study validates a human PD1‐CD137L fusion protein as a potent drug candidate for cancer immunotherapy.

## Graphical abstract

A PD1‐CD137L fusion protein converts the immunoinhibitory PD‐L1 to an immunostimulatory signal and is more effective than targeting PD‐L1 and CD137 separately.

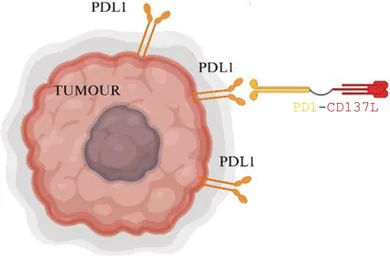

## 1 Introduction

Despite impressive efficacies in blood cancers, the success of immune checkpoint inhibitors (ICIs) in solid tumours remains limited [1]. ICIs alone often fail because T cells inside solid tumours are insufficiently activated. Therefore, removing immune inhibition remains ineffective. Immune stimulators alone often fail since T cell activation is negated by the immunosuppressive nature of the tumour microenvironment (TME). However, the combination of ICI with immune activators targets both mechanisms, and synergistically leads to T cell activation that can eliminate malignant cells [2, 3]. This is the reason why combinations of ICIs that prevent inhibitory signals with agents that promote activation of T cells are being explored. CD137 (4‐1BB, TNFRSF9), a T cell costimulatory molecule is inducibly expressed on T cells and natural killer (NK) cells. Upon cognate interaction with its ligand, CD137L, on antigen presenting cells (APC), CD137 multimerises and recruits the adaptor proteins TNF receptor‐associated factors TRAF1 and TRAF2, which form the nucleation core for downstream signalling. Subsequent activation of the canonical NF‐κB signalling cascade drives the transcription of genes essential for T cell activation, proliferation, cytokine production (e.g., IL‐2, IFN‐γ) and resistance to apoptosis [4, 5].

Agonistic antibodies targeting CD137 are being tested in clinical trials for tumour immunotherapy, among which urelumab and utomilumab are most advanced. However, as a monotherapy, urelumab was associated with dose‐dependent hepatotoxicity, whereas utomilumab demonstrated limited anti‐tumour efficacy [6]. A potential solution to prevent toxicity may be the use of lower doses in combination therapies.

In order to target PD‐L1 and CD137, bispecific antibodies have been developed including MCLA‐145, GEN1046 and IBI319 which are currently being evaluated in preclinically and clinically [7, 8, 9]. We hypothesised that using the natural ligands for PD‐1 and CD137 instead of antibodies may result in a more effective T cell activation. Here we report a recombinant bispecific fusion protein that combines the extracellular domain of PD‐1 with the extracellular domain of CD137L (hPD1‐CD137L). It binds to and neutralises the PD‐L1 in the TME and converts this immunoinhibitory signal to the T cell costimulatory CD137L signal. PD1‐CD137L protein is stable over time and at low pH as found in the TME. It costimulates T cells of healthy donors and of tumour infiltrating lymphocytes (TILs) and enhances T cell‐mediated killing of a range of cancer cell lines and of tumour organoids. PBMC‐humanised NSG mice showed no toxicity in response to hPD1‐CD137L. Efficacy study using PBMC‐humanised, Rd18 or C666‐engrafted NSG mice demonstrated that hPD1‐CD137L can suppress tumour growth without eliciting overt toxicity.

## 2 Material and Methods

### 2.1 Cell Lines

Lung carcinoma cell line A549, nasopharyngeal carcinoma (NPC) cell line C666, and rhabdomyosarcoma (RMS) cell line Rd18 were cultured in RPMI 1640 medium supplemented with 10% FBS and 1% streptomycin and penicillin. HEK293 cells were cultured in DMEM medium supplemented with 10% FBS and 1% streptomycin and penicillin. All cells were cultured in a humidified 37°C and 5% CO_2_ incubator. Cell lines have been obtained from ATCC (Manassas, Virginia, USA).

### 2.2 Isolation of Peripheral Blood Mononuclear Cells (PBMCs) and CD3 ^+^ T Cells

All human blood samples were obtained from healthy donors at Health Sciences Authority Singapore with approval from the Institutional Review Board, NUS, Singapore (IRB number: 13‐079E) in accordance with the guidelines of the Health Sciences Authority of Singapore. PBMCs were isolated via density gradient centrifugation using Lymphoprep (STEMCELL, #07851) followed by removal of red blood cells with the Ammonium‐chloride‐potassium (ACK) lysis buffer. The isolated PBMCs were resuspended in PBS containing 2% FBS for direct use or for isolation of T cells using the EasySep human T cell isolation kit (STEMCELL Technologies). All primary cells were cultured in RPMI 1640 medium supplemented with 10% fetal bovine serum (FBS), 1% streptomycin and penicillin (R10‐PS) and 100 IU/mL IL‐2 in a humidified 37°C and 5% CO_2_ incubator.

### 2.3 Recombinant Protein Design, Production and Purification

hPD1‐CD137L was engineered with the PD1 ectodomain (NM_005018.3) at N‐terminus and CD137L ectodomain (NM_003811) at C‐terminus, separated by a 6 × His tag with flexible linkers. Recombinant CD137L served as control. A pYD7‐hPD1‐CD137L plasmid was transiently transfected into HEK293‐6E suspension cells using the PEI method. Five days later, cultures were centrifuged and supernatants were incubated with nickel (Ni) resin overnight (4°C). Resin was washed (Tris/NaCl with 5 mM imidazole) with protein eluted (500 mM imidazole), buffer‐exchanged into PBS (10 kDa MWCO), and quantified by NanoDrop A280.

### 2.4 Coomassie Blue Staining and Western Blot for Protein Characterisation

Proteins were characterised by SDS–PAGE and Western blot analysis. For denaturing SDS–PAGE, samples were mixed with 5xLaemmli buffer containing 2.5% β‐mercaptoethanol, boiled (99°C, 5 min), and run on 10% gels (100 V, 2–3 h). Gels were Coomassie‐stained (InstantBlue, 15 min), rinsed, and imaged (ChemiDoc). Monomer size was confirmed by anti‐His blotting (BioLegend, clone J095G46; 1:1000) with HRP anti‐mouse IgG secondary antibody (Life Technologies, clone G21040; 1:10000) and chemiluminescent substrates. Multimers were assessed by omitting β‐mercaptoethanol and probing with anti‐human CD137L (BioLegend, clone 5F4; 1:1000) antibody.

### 2.5 Flow Cytometry for Binding Assays

To test the binding of the PD1 or CD137L domain of human fusion protein, PD‐L1‐expressing cells or CD137‐expressing cells were incubated with 10 μg/mL of fusion protein for 1 h in a humidified 37°C and 5% CO_2_ incubator. After 1 h, the cells were stained with LIVE/DEAD Fixable Near‐IR Dead Cell Stain Kit (Life Technologies) in the dark for 30 min at room temperature. This was followed by staining with anti‐human PD‐L1 antibody (Invitrogen, clone MIH3), anti‐His‐tag antibody (Biolegend, clone J095G46), anti‐human CD137L antibody (Biolegend, clone 5F4) and anti‐human PD‐1 antibody (Biolegend, clone EH12.2H7) in the dark for 30 min at 4°C. Measurement was done on LSRFortessa X‐20 flow cytometer.

### 2.6 Western Blot to Elucidate NF‐kB Pathway in T Cells

T cells were isolated and activated on immobilised anti‐human CD3 (Biolegend, clone OKT3, 1 μg/mL) under indicated treatments. After 24 h, cells were washed and lysed in RIPA buffer on ice (10 min), centrifuged (10 000 rpm, 10 min) and supernatants stored at −20°C. Protein was quantified by Bradford assay. Lysates (30 μg) were prepared in Laemmli buffer with 2.5% β‐mercaptoethanol, boiled (99°C, 5 min), run on 10% SDS–PAGE and transferred to PVDF. Membranes were blocked (5% milk/TBST) and probed for phospho‐p65, GAPDH, then stripped and reprobed for total p65; bands were detected by ChemiDoc.

### 2.7 Flow Cytometry for T Cell Status Evaluation

T cells were isolated and activated on immobilised anti‐human CD3 (OKT3, 1 μg/mL) for 24 h and treated with indicated conditions for another 24 h. The cells were then harvested and processed with Fixable Near IR live/dead dye, FCR blocker (Miltenyi Biotec) and antibody cocktail. For extracellular staining, cells were stained with the following cell surface markers: CD3 (Biolegend, clone: UCHT1), CD4 (Biolegend, clone: SK3), CD8 (Biolegend, clone: SK1), CD69 (Biolegend, clone: FN50), HLA‐DR (Biolegend, clone: G46‐6), PD1 (Biolegend, clone: EH12.2H7) and LAG‐3 (Biolegend, clone: 11C3C65). For intracellular staining, cells were activated with a master mix containing PMA (Sigma, 50 ng/mL), ionophore (Sigma, 500 ng/mL) and Brefeldin A (Invitrogen, 100 ×) for 4 h before being processed for flow cytometry. IFN‐γ (Biolegend, clone: B27), TNF (Biolegend, clone: MAb11) and Granzyme B (Biolegend, clone: QA16A02) were stained.

### 2.8 In Vitro Coculture Assays

PD1‐CD137L‐mediated enhancement of effector function was assessed in 2 dimensional (2D) and 3D cocultures using real‐time cytolysis readouts. For 2D assays, CD3^+^ T cells were purified from healthy‐donor PBMCs and activated with immobilised anti‐CD3 (OKT3, 1 μg/mL) for 24 h. In parallel, tumour cells were seeded overnight in xCELLigence E‐plates. Activated T cells were then added at defined effector:target ratios with or without fusion protein (10 μg/mL; conditions in duplicate). Tumour killing was quantified as changes in impedance (cell index) converted to % cytolysis.

In 2D assays with TILs, expanded TILs, derived from treatment‐naïve NPC tumour biopsies, were used. Briefly, 2–3 pieces of NPC tumour fragments of 2–3 mm^3^ were dissociated using human tumour dissociation kit (Miltenyi Biotec). The resulting cell‐suspension is further filtered through 70 μM cell strainer to remove any remaining clumps. Following which, the initial TIL expansion was initiated in 24‐well plates; TILs were expanded for 2 weeks using 150 IU/mL recombinant interleukin‐(rhIL‐2; Proleukin) and TransACT (anti‐CD3 and anti‐CD28 antibodies, Miltenyi Biotec) using TexMACs Media (Miltenyi Biotec). Rapid expansion protocol (REP) of TILs was then applied to 2 weeks‐old expanded TILs, where they were stimulated further with anti‐CD3 OKT3, 3000 IU/mL rhIL‐2 and 50‐Gy irradiated allogenic PBMCs at 1:100 E:T ratio for another 2 weeks in TexMACs media in T175 flasks.

For 3D assays, colorectal cancer (CRC) tumour organoids were derived from fresh CRC tumour tissue [10]. The tissues were cut into smaller fragments, minced and further processed to obtain the tumour dissociates. The tumour dissociates were then resuspended in 200 μL Cultrex UltiMatrix Reduced Growth Factor Basement Membrane Extract (BME) (R&D Systems, #BME001‐05) and seeded as 8–10 domes in a well of a warmed 6‐well plate. The plate was inverted and incubated in a 37°C incubator for 20 min. Once BME was polymerised, 2 mL CRC organoids culture media, supplemented with 10 μM Y‐27632, were added. The tumour dissociates were cultured with media change once every 2 days. After 10 days, the developed organoids were passaged into a new plate (P1). The organoids were continually passaged until P5. CRC organoids of passage 5 were used for the experiment.

CD3^+^ T cells were activated with immobilised OKT3 (1 μg/mL, 24 h). Patient‐derived colorectal cancer organoids were labelled with CellTracker Green and primed with IFN‐γ (200 ng/mL, 24 h), then seeded at 10,000 cells/well. T cells were added at 5:1 (E:T) in RPMI +10% FBS + 1% penicillin/streptomycin with propidium iodide to label dead cells. Conditions included medium only, 10 μg/mL rCD137L + IgG1, rCD137L + PD‐L1 antibody or hPD1‐CD137L (technical duplicates; two donors). Plates were imaged every 45 min (Incucyte S3; red/green channels) for 48 h; % cytolysis was calculated as (red+green)/green cells.

### 2.9 In Vivo Humanised Mouse Model

To assess systemic toxicity, six 7‐week‐old male NSG mice (InVivos) were randomised to saline or hPD1‐CD137L. Each mouse received 10^7^ human PBMCs intravenously (i.v.) on Day 1. From Day 4, mice were dosed i.v. with saline or 10 mg/kg hPD1‐CD137L every 3 days (Days 4, 7 and 10). Body weight and survival were monitored daily. Four days after the final dose (Day 14), cheek blood was collected for serum, mice were euthanised and livers harvested. Human IFN‐γ and TNF were quantified in serum and liver homogenates by ELISA (R&D Systems; Invitrogen) per manufacturer's instructions.

To evaluate efficacy, Rd18 and C666 models were established separately. For Rd18 model, 3 × 10^6^ cells were mixed with Matrigel (Corning) in a 1:1 ratio and injected subcutaneously (s.c.) in the right flank of ten 7‐week‐old male NSG mice (Day 1). When tumours became palpable, the mice were then reconstituted with 10^7^ human PBMCs i.v. (Day 9). From Day 12, mice received saline or 10 mg/kg hPD1‐CD137L i.v. every 3–4 days for six doses. Body weight and tumour volume were measured daily (0.5 × L × W^2^). On Day 30, tumours were excised, weighed and measured; groups were compared by unpaired *t*‐test. For C666 model, ten 7‐week‐old male NSG mice were first reconstituted with 10^7^ human PBMCs (Day 1) before being engrafted with 10^6^ C666 cells (Day 10). From Day 15, mice received saline or 10 mg/kg hPD1‐CD137L i.v. every 3 days for four doses. Body weight and tumour volume were measured 2–3 times every week. On Day 33, tumours were excised, weighed and measured; groups were compared by unpaired *t*‐test.

### 2.10 Statistical Analysis

Statistical evaluation was performed using GraphPad Prism 9.0 (GraphPad Software). Statistical significance was determined by Student's *t*‐test, one‐way ANOVA with multiple comparisons.

## 3 Results

### 3.1 Production of Recombinant Human PD1‐CD137L

In the human PD1‐CD137L (hPD1‐CD137L) fusion protein, the extracellular domain of PD1 (exPD1) is placed at the N‐terminus whereas the extracellular domain of CD137L (exCD137L) is placed at the C‐terminus (Figure 1A) because PD‐1 is a Type I transmembrane protein which naturally possesses the extracellular domain at its N terminus, while CD137L, as a type II transmembrane protein, has the extracellular domain at its C terminus [11, 12]. To ensure efficient secretion from transfected HEK293‐6E cells, the PD‐1 signal peptide was added upstream of the PD‐1 extracellular domain. A His‐tag was inserted between the PD‐1 and CD137L domains, flanked by short glycine‐serine linker sequences to provide spatial separation. This separation reduces the chance of the His‐tag being buried within the 3‐dimensional conformation of the fusion protein, thereby facilitating its interaction with nickel (Ni^2+^) beads for optimal purification.

**FIGURE 1:**
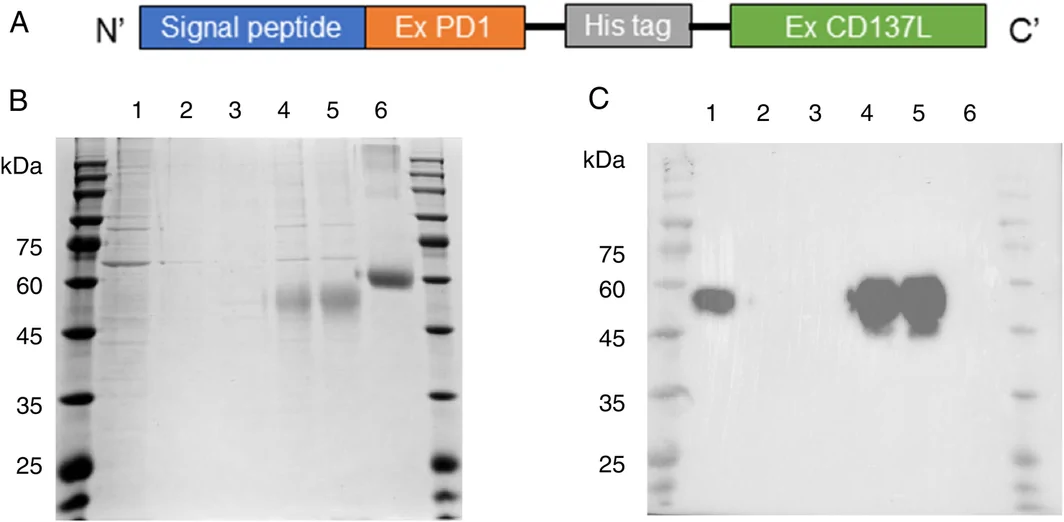
Expression of recombinant human PD1‐CD137L. (A) Structural composition of hPD1‐CD137L. (B) Coomassie blue staining of flowthroughs collected during protein purification step. (C) Western blot against His‐tag. Legend for (B, C): Lane 1: Supernatant flowthrough; Lane 2: Equilibration step flowthrough; Lane 3: Wash step flowthrough; Lane 4: Purified protein after elusion and buffer exchange; Lane 5: Purified protein from previous batch (positive control); Lane 6: Bovine serum albumin (negative control).

To evaluate the purity and molecular size of hPD1‐CD137L, flow‐throughs collected during the purification process were separated by denaturing SDS‐PAGE and visualised by Coomassie blue staining which revealed a prominent band at approximately 55 kDa, corresponding to the expected molecular weight of hPD1‐CD137L with post‐translational modification (Figure 1B). A Western blot analysis with anti‐His‐tag primary antibody corroborated the identity of the expected fusion protein (Figure 1C).

### 3.2 The PD‐1 and CD137L Domains Bind Their Native Targets

The binding specificity of the PD1 domain of hPD1‐CD137L was tested by incubating Rd18 RMS cells, which naturally express high levels of PD‐L1, with hPD1‐CD137L. Medium only or BSA were used as negative controls to confirm true binding of fusion protein to PD‐L1 (Figure 2A). Among PD‐L1‐positive Rd18 cells, the three domains of the fusion protein (PD1, His‐tag and CD137L) were observed exclusively following incubation with hPD1‐CD137L (Figure 2B–D).

**FIGURE 2:**
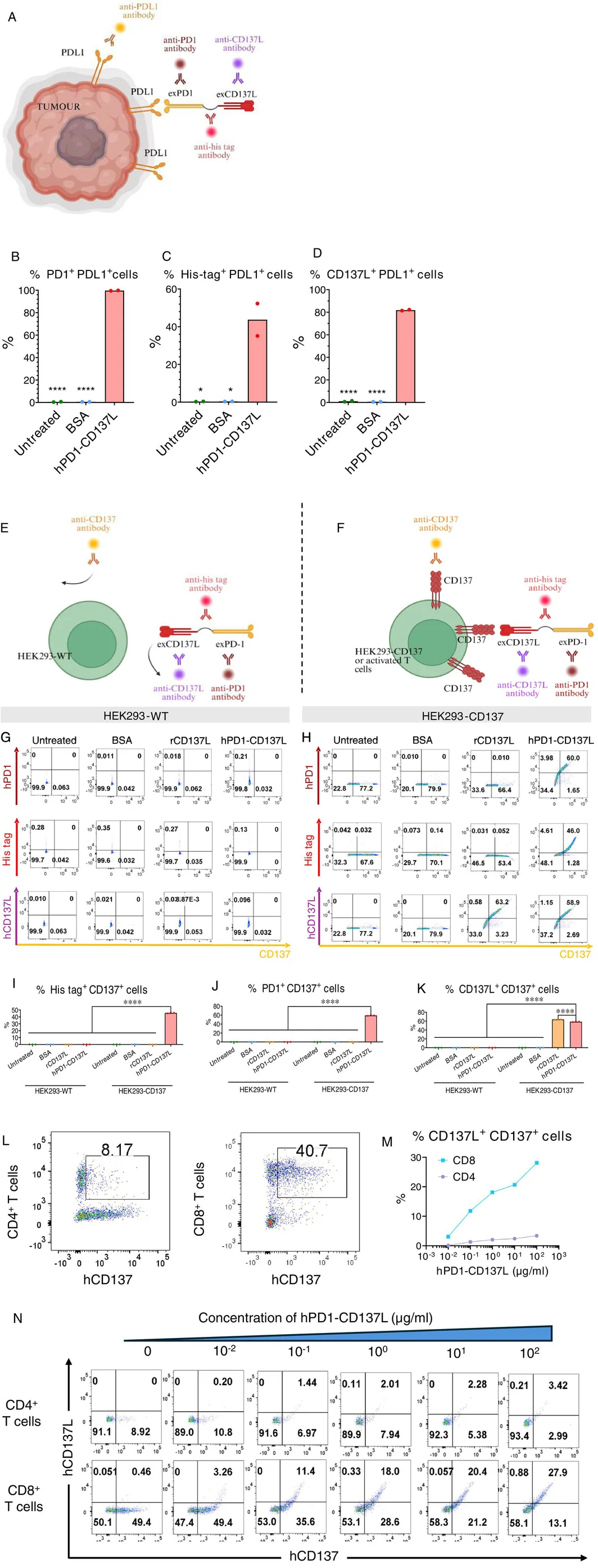
Both PD1 and CD137L domains can bind to their respective native targets. (A) Schematic depiction of the test method of the PD1 domain. (B–D) Compiled percentages of the double positive (%) population in Rd18 cells upon incubation with indicated proteins. The experiment was done in duplicates and analysed using ordinary one‐way ANOVA. (E, F) Schematic depiction of the test method of the CD137L domain. (G, H) Representative scatter plots showing distribution of HEK293‐WT and HEK293‐CD137 cells under different treatment conditions. (I–K) Compiled percentages (%) of the double positive populations in HEK293‐WT and HEK293‐CD137 cells upon incubation with different proteins. hPD1‐CD137L, rhCD137L and BSA were used at 10 μg/mL. The experiment was done in triplicates and analysed using two‐way ANOVA. (L) CD137 expression in CD4^+^ and CD8^+^ T cells in one representative donor. (M) Binding specificity of CD137L domain to CD4^+^ and CD8^+^ T cells at different concentrations. (N) Representative scatter plot showing the distribution of T cells upon incubation with different concentrations of hPD1‐CD137L.

Similarly, cells that express CD137 but not PD‐L1 were used to verify the binding specificity of the CD137L domain. CD137‐deficient HEK293‐wildtype (WT) cells and CD137‐transfected HEK293‐CD137 cells were incubated with BSA (negative control), recombinant CD137L (rCD137L, positive control) or hPD1‐CD137L (Figure 2E,F). HEK293‐WT cells did not bind to rCD137L or hPD1‐CD137L, while HEK293‐CD137 cells were stained positive for human PD1, His‐tag and human CD137L (Figure 2G–K). This confirms the structural intactness of the CD137L domain and its binding specificity for CD137. The positive control, rCD137L, also stained only HEK293‐CD137 cells (Figure 2G–K). T cells express CD137 upon activation, with higher levels found on CD8^+^ T cells (Figure 2L). Incubation with exponentially increasing concentrations of hPD1‐CD137L showed that both activated CD4^+^ and CD8^+^ T cells bind hPD1‐CD137L dose‐dependently when assayed for the presence of the CD137L (Figure 2M,N).

### 3.3 hPD1‐CD137L Forms Monomers and Trimers That Are Stable Over Time and at Low pH

SDS‐PAGE and Western blot analysis of hPD1‐CD137L revealed three distinct bands, suggesting that it exists in monomeric, trimeric and higher‐order polymeric forms (Figure S1A). While the higher‐order forms are a small minority, the monomeric form dominates, with the amount of the trimeric form of hPD1‐CD137L being 2/3 of the monomeric form (Figure S1B). On a reducing SDS‐PAGE, only the monomeric form was visible, indicating that disulphide bonds are required for the formation of trimeric and higher‐order multimeric states (Figure S1C).

To assess the structural stability of hPD1‐CD137L over time, aliquots were stored at 4°C for 3, 6, 8 and 11 months. All aliquots retained structural integrity, demonstrated by the PD1 and CD137L domains displaying similar binding capacity across different storage durations (Figure S1D,E).

The tumour microenvironment (TME) commonly exhibits an acidic extracellular pH, usually between 6.0 and 6.8, which is substantially lower than the physiological pH of approximately 7.4. hPD1‐CD137L bound to Rd18 cells via its PD1 domain even after reducing the pH to 5.9 with lactic acid (LA) (Figure S1F–H).

There may be unfolding of the His‐tag and CD137L domain as pH decreases, resulting in the exposure of epitopes of His‐tag and CD137L for the respective antibodies to access and detect (Figure S1G,H). This would explain the increased signals at lower pH. The unfolding was not as evident for the PD1 domain, likely due to its binding to PD‐L1 on Rd18 cells which may have stabilised the structure of the PD1 domain.

Similarly, the binding of the CD137L domain to CD137 on HEK293 cells only slightly increased with decreasing pH (Figure S1I–K), which also suggests that the CD137L domain is stabilised upon engagement with its cognate receptor on HEK293 cells (Figure S1N), while that was not observed using the CD137‐negative Rd18 cells (Figure S1H). Furthermore, the multimeric status of hPD1‐CD137L was conserved across all pH values tested (Figure S1L), likely due to the strong covalent disulphide bonds that hold the multimers of the fusion protein together.

To assess whether the PD1‐CD137L fusion protein interferes with binding of anti‐PD‐L1 antibody and to get an estimate of its affinity, we performed competitive binding assays of PD1‐CD137L protein and the anti‐PD‐L1 antibody atezolizumab, and analysed the levels of PD1‐CD137L and atezolizumab by flow cytometry (Figure S2A,B). It made a difference whether PD‐L1‐expressing Rd18 cells were first incubated with increasing concentrations of PD1‐CD137L fusion protein and then with atezolizumab (Figure S2C–G), or in the reverse order (Figure S2H–L), before cells were washed and stained with anti‐human IgG to detect binding of atezolizumab or with anti‐PD1, anti‐CD137L or anti‐His to detect the PD1‐CD137L fusion protein or with anti‐PD‐L1 to detect free PD‐L1.

The presence of PD1‐CD137L fusion protein, even at the highest concentration of 20 μg/mL did not prevent binding of atezolizumab to PD‐L1 on Rd18 cells (Figure S2A–C), while atezolizumab, at its highest concentration (20 μg/mL) almost completely displaced binding of PD1‐CD137L, as shown for the absence of CD137L, PD‐1 and HIS staining (Figure S2E–G). At intermediate concentrations of 2, 5 or 10 μg/mL of atezolizumab, increasing concentrations of PD1‐CD137L led to correspondingly higher staining of CD137L, PD1 and HIS (Figure S2E–G).

However, when atezolizumab was added first, PD1‐CD137L protein could not displace it, independent of the concentrations used (range 2–20 μg/mL) (Figure S2H). Rather, binding of PD1‐CD137L protein was considerably lower than when we added it first (Figure S2E–G vs. J–L). These data indicate that PD1‐CD137L protein and atezolizumab bind to overlapping epitopes, and that atezolizumab has a higher affinity for PD‐L1 than the PD1‐CD137L protein.

PD‐L1 expressed on Rd‐18 cells remained detectable by the anti‐PD‐L1 antibody used (clone MIH3) in the presence of low concentrations of atezolizumab. However, MIH3 binding progressively decreased with increasing concentrations of atezolizumab and was almost completely abolished at 20 μg/mL, suggesting that MIH3 and atezolizumab recognise overlapping or closely adjacent regions on PD‐L1 (Figure S2D,I).

### 3.4 hPD1‐CD137L Enhances Activation of T Cells via the NF‐κB Signalling Pathway

The co‐stimulatory receptor CD137 is expressed on activated T cells, and the induction of CD137 signalling by its ligand, CD137L, or by an agonistic anti‐CD137 antibody enhances T cell activation. Specifically, CD137 recruits TNF receptor‐associated factors (TRAFs), particularly TRAF1 and TRAF2, which initiate the activation of the nuclear factor kappa‐light‐chain‐enhancer of activated B cells (NF‐κB) pathway, along with JNK and p38 MAPK pathways [5, 13, 14]. Among these, the NF‐κB pathway is a critical mediator of CD137‐induced immune enhancement, promoting transcription of genes associated with T cell survival, cytokine production (e.g., IL‐2, IFN‐γ) and anti‐apoptotic responses [15]. Upregulation of phospho‐p65 is a hallmark of NF‐κB activation and thus was used as a readout to demonstrate that hPD1‐CD137L is able to initiate CD137 downstream signalling [16].

To assess whether the human PD1‐CD137L fusion protein activates the NF‐κB signalling pathway, Western blot analysis was performed to detect total and phosphorylated NF‐κB p65 in primary T cells. GAPDH was probed as a loading control. Given that CD137 is inducibly expressed upon T cell activation, primary T cells were first stimulated with an anti‐CD3 antibody and then cultured for 24 h under different conditions. T cells treated with soluble hPD1‐CD137L exhibited a clear increase in phospho‐p65 (Ser536) band intensity compared to untreated controls, indicating activation of the canonical NF‐κB pathway (Figure 3A). Total p65 levels remained unchanged, confirming that the observed increase was due to phosphorylation rather than overall protein upregulation. Densitometric analysis revealed a 2.8‐fold increase in phospho‐p65 signal normalised to GAPDH when pre‐activated T cells were incubated with soluble hPD1‐CD137L (Figure 3B). These findings suggest that the CD137L domain in the fusion protein successfully stimulated CD137 on primary T cells, activating the downstream NF‐κB pathway that results in the transcription of T cell activation and survival genes.

**FIGURE 3:**
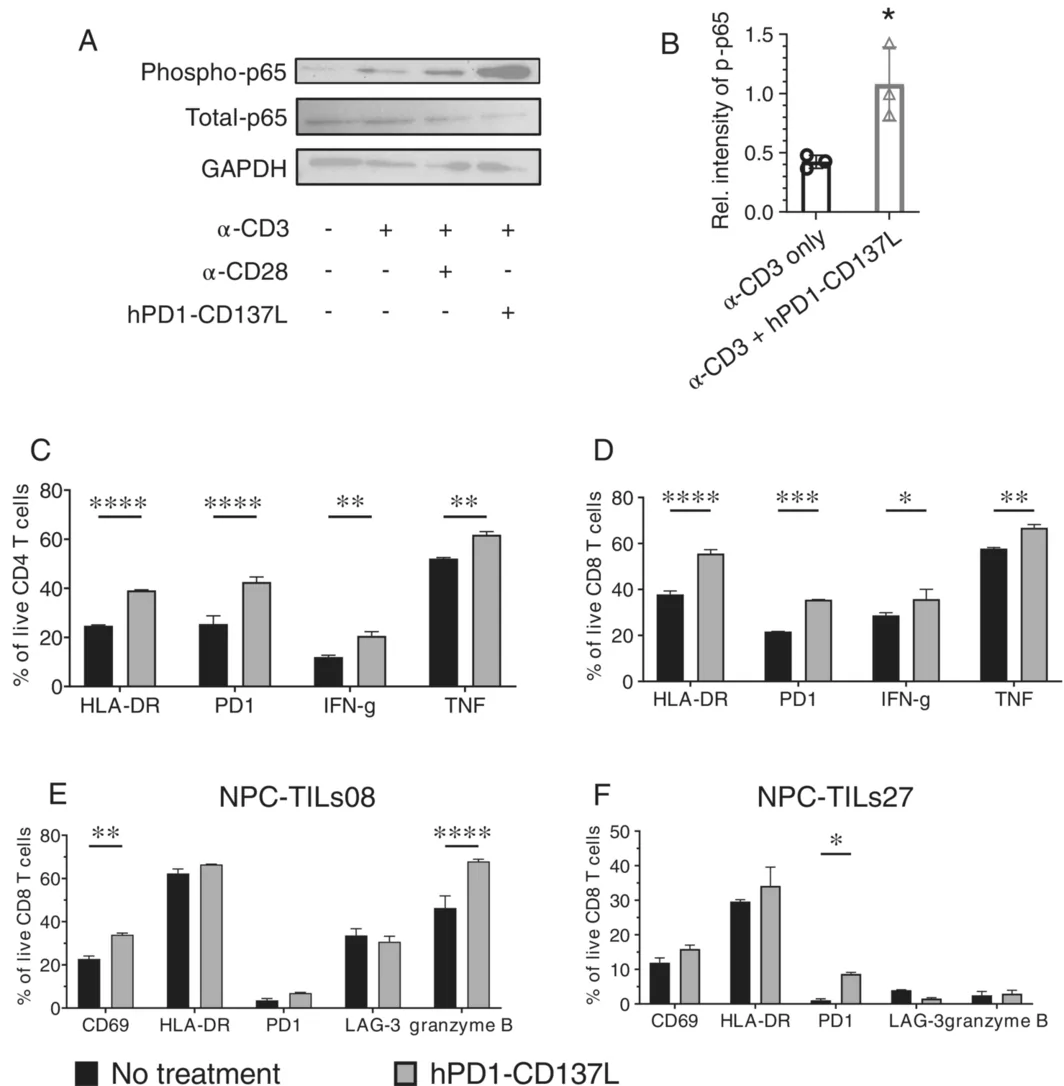
CD137L domain is functional in promoting T cell activation. (A) p65 intensity upon T cell stimulation by different conditions in one representative donor. (B) Compiled bar chart showing the relative band intensity of phospho‐p65 normalised to GAPDH in T cells activated with an a‐CD3 antibody only and with the addition of hPD1‐CD137L at 10 μg/mL. in 3 independent healthy donors as quantified by ImageJ and analysed using unpaired *t*‐test in Prism. (C, D) The activation status of CD4 and CD8 T cells upon incubation with hPD1‐CD137L. The experiment was done in duplicates for each donor and repeated with T cells from 2 independent healthy donors. (E, F) Activation status of CD8 T cells of 2 NPC‐patient derived TILs. NPC‐TIls, Tumour infiltrating lymphocytes from nasopharyngeal carcinoma patients.

The immunomodulatory effects of hPD1‐CD137L were further assessed by immunophenotyping primary T cells of healthy donors as well as patient‐derived tumour‐infiltrating lymphocytes (TILs) (Figure 3 and Figure S3). A panel of surface activation markers and intracellular cytokines was analysed. In T cells of healthy donors, hPD1‐CD137L increased expression of HLA‐DR, PD‐1, IFN‐γ and TNF on both CD4^+^ and CD8^+^ T cell populations compared to the untreated controls (Figure 3C,D). The upregulation of HLA‐DR together with the concomitant elevated production of the pro‐inflammatory cytokines IFN‐γ and TNF, indicates that hPD1‐CD137L enhances and sustains T cell activation and cytotoxic effector function, suggesting its potential to augment anti‐tumour immune responses. Increased PD‐1 expression may indicate T cell exhaustion but also T cell activation [17]. Further, it cannot be ruled out that increased PD‐1 levels are not only due to increased expression of PD‐1 by T cells but due to hPD1‐CD137L binding to CD137 on activated T cells. In TILs from NPC patients, only CD8^+^ T cells were analysed due to near complete absence of CD4^+^ T cells among the ex vivo‐expanded patient‐derived TILs. The two NPC‐derived TIL populations displayed markedly distinct baseline immune phenotypes (Figure 3E,F). Without any treatment, NPC‐TILs08 exhibited a higher activation status than NPC‐TILs27, as evidenced by higher CD69 (an early activation marker), HLA‐DR (a late activation marker) and granzyme B (a key cytolytic effector protein) levels. Following hPD1‐CD137L treatment, CD8^+^ T cells from the NPC‐TILs08 donor showed increased expression of CD69, elevated granzyme B, and a slight reduction in LAG‐3, an inhibitory receptor associated with T cell exhaustion (Figure 3E). In CD8^+^ T cells from the NPC‐TILs27 donor, hPD1‐CD137L induced an increase in CD69 and PD‐1 expression, as well as a slight decrease of LAG‐3 (Figure 3F). Collectively, despite the donor‐specific differences in immunophenotype, both at baseline and following hPD1‐CD137L exposure, these observations indicate that hPD1‐CD137L exerts an immunostimulatory effect capable of enhancing activation and potentially restoring or augmenting the anti‐tumour effector functions of CD8^+^ T cells within patient‐derived TIL populations.

### 3.5 hPD1‐CD137L Promotes Tumour Cell Cytolysis in 2D and 3D Cocultures

After verifying the binding specificity and structural integrity of the human PD1‐CD137L protein and ensuring that the CD137L domain induced the NF‐κB pathway in T cells, 2D and 3D coculture assays were used to assess the anti‐tumour effect of the hPD1‐CD137L.

For the 2D model, three PD‐L1‐expressing cancer cell lines, namely Rd18 (RMS), C666 (NPC) and A549 (Lung Carcinoma) were used as target cells in cocultures with effector T cells. For effector cells, CD3^+^ T cells isolated from healthy donors' peripheral blood mononuclear cells (PBMCs) were employed for all three tumour cell lines, while tumour infiltrating lymphocytes (TILs) from NPC patients were available for the NPC cells C666. To monitor tumour cell death in the 2D coculture, the xCELLigence Real‐Time Cell Analysis (RTCA) system was used to provide a real‐time, label‐free and impedance‐based tumour cytolysis measurement. Area under the graph of % cytolysis was computed for meaningful comparison. The potency of hPD1‐CD137L was assessed in three ways as follows [1]: compared to the untreated control [2], compared to single agent therapy where only the PD1/PD‐L1 or the CD137/CD137L pathway was targeted, and [3] compared to combination therapy where both pathways were targeted.

Comparison to the untreated control: As shown in the individual cytolysis curves, T cells from healthy donors induced a time‐dependent tumour cell death in all three tumour cell lines, reflected by a progressive increase in cytolysis over coculture time (Figure S4). The statistically significant difference in their complied areas under % cytolysis curves (Figure 4A,F,K) indicates that hPD1‐CD137L enhanced the cytotoxic effector functions of T cells. However, the extent of the cytolysis enhancement and its kinetics varied from donor to donor, which could be due to different CD4^+^/CD8^+^ T cell ratios among other factors. Some cytolysis curves reached a maximum plateau during the first 20 h of coculture while others reached the peak after 40 h (Figure S4). Nevertheless, the reproducibility across multiple donor‐derived T cells highlights the robustness and donor‐independent potential of hPD1‐CD137L in enhancing immune‐mediated tumour cell killing.

**FIGURE 4:**
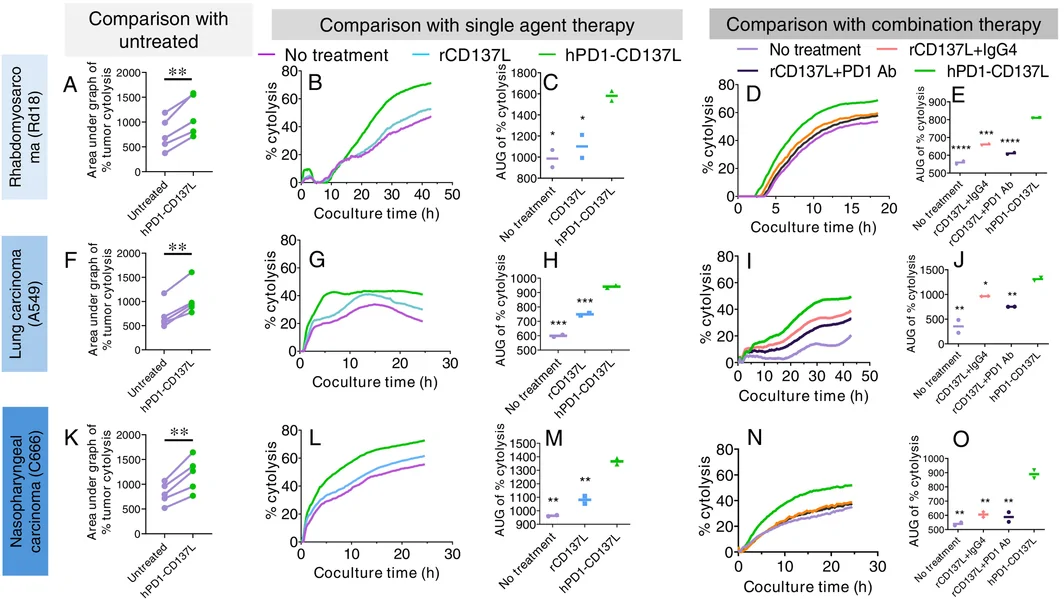
hPD1‐CD137L promoted tumour cytolysis in 2D coculture assays in vitro. (A, F, K) The areas under the individual % cytolysis across 5 donors' T cells as compiled in Prism and analysed by paired *t*‐test. (B, C, G, H, L, M) Performance of hPD1‐CD137L compared to single agent therapy in one representative donor. (D, E, I, J, N, O) Performance of hPD1‐CD137L compared to combination therapies in one representative donor. Anti‐PD‐L1 antibody (anti‐PD‐1, Pembrolimumab), hPD1‐CD137L and rhCD137L were used at 10 μg/mL. The experiment was conducted using 2 donors' T cells with technical duplicates and at an effector‐to‐target ratio of 6.7:1. Statistical analysis was done in Prism using ordinary One‐way ANOVA. rCD137L: Recombinant CD137L protein; AUG, Area under % cytolysis graph.

Comparison with single agent therapy: The hPD1‐CD137L was compared to recombinant human CD137L protein (rCD137L), which solely engages the CD137 co‐stimulatory receptor. As expected, CD137L enhanced the cytolytic activity of T cells, but hPD1‐CD137L proved to be more potent (Figure 4B,C,G,H,L,M). This is likely because hPD1‐CD137L not only provides costimulation to T cells but also releases immune inhibition by PD‐L1. This dual function resulted in a more robust immune‐mediated cytotoxicity, as reflected in the increased target cell lysis.

Comparison with combination therapy: Finally, we comparatively evaluated the potency of the PD1‐CD137L fusion protein to targeting CD137 and PD‐L1 separately. pembrolizumab, a clinically approved anti‐PD1 antibody was used to block PD‐1 on T cells, and rCD137L protein was used to engage with CD137. hPD1‐CD137L consistently stimulated T cells to a higher cytolysis than the combination therapies of rCD137L and anti‐PD‐1 antibody (Figure 4D,E,I,J,N,O). This is likely due to different modes of action. It is possible that the PD1‐CD137L fusion protein provides a more spatially coordinated and simultaneous delivery of both signals to T cells, while two separate agents, albeit given at the same time, may result in many or some T cells only receiving one signal, or both signals at different times, leading to only a partial activation.

To assess the performance of hPD1‐CD137L in more clinically relevant models, TILs derived from two NPC patients were used as effector cells in 2D coculture assays with C666 cells. Since TILs have been naturally primed and stimulated within the TME, they exhibit a baseline level of functional readiness, as evidenced by moderate expression of activation markers CD69 and HLA‐DR (Figure 3E,F). As a result, lower effector‐to‐target (E:T) ratios were used in the coculture assays. hPD1‐CD137L‐treated TILs more effectively promoted cytolysis of C666 NPC cells than untreated or rCD137L‐treated TILs (Figure 5A–H). The results were reproducible with both donors' TILs and at two different E:T ratios, demonstrating the potency of hPD1‐CD137L in amplifying TIL‐mediated cytotoxicity.

**FIGURE 5:**
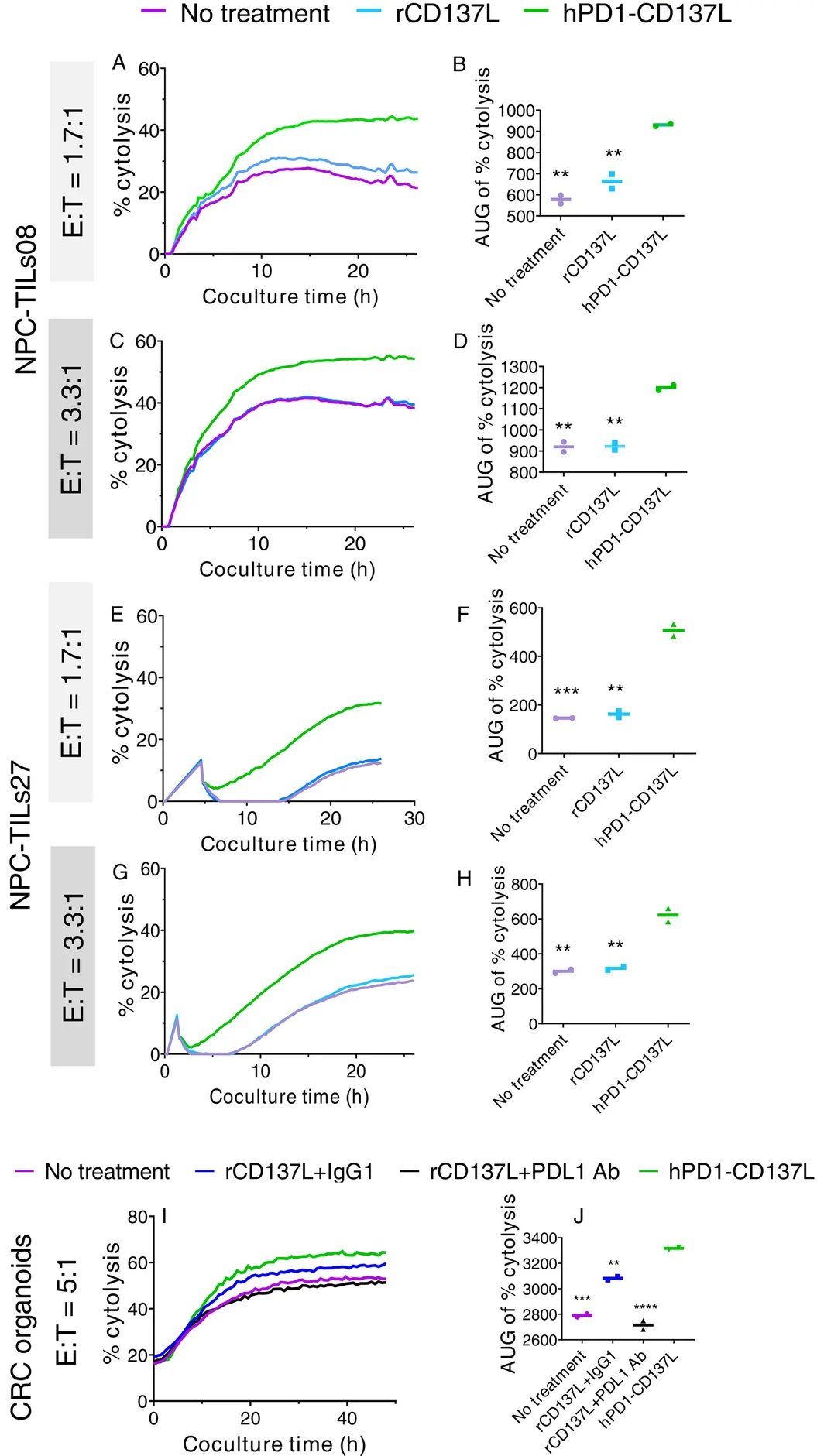
hPD1‐CD137L enhanced tumour cytolysis in more clinically relevant models. (A–D) Real time % cytolysis of C666 and its area under graph using E:T ratios of 1.7:1 and 3.3:1 for the donor NPC‐TILs08. (E–H) Real time % cytolysis of C666 and its area under graph using E:T ratios of 1.7:1 and 3.3:1 for the donor NPC‐TILs27. (I, J) Cytolysis of patient‐derived colorectal tumour organoid when cocultured with one representative healthy donor’ T cells at different treatment conditions. Anti‐PD‐L1 antibody (anti‐PDL1, atzeolizumab), hPD1‐CD137L, rhCD137L and IgG1 were used at 10 μg/mL. The experiment was conducted using 2 donors' T cells with technical duplicates and at an effector‐to‐target ratio of 5:1. Statistical significance was computed in Prism using one‐way ANOVA. AUG, Area under graph of % cytolysis; CRC, Colorectal cancer.

We also employed a 3D tumour organoid, derived from colorectal cancer patients in cocultures with T cells of 2 different healthy donors. A PD‐L1 antibody (clone atezolizumab) was added together with rCD137L for comparing the hPD1‐CD137L fusion protein to inhibiting PD‐L1 and costimulating CD137 separately. hPD1‐CD137L induced higher tumour cell killing with T cells of both donors (Figure 5I,J shows a colorectal cancer derived from a Stage 2a, primary caecum adenocarcinoma). These findings indicate that hPD1‐CD137L could have a higher therapeutic potential than conventional dual‐agent combinations.

### 3.6 hPD1‐CD137L Fusion Protein Is Safe and Efficacious in Murine Tumour Models in Humanised Mice

In preparation for in vivo efficacy testing, we assessed the potential in vivo toxicity of hPD1‐CD137L. To investigate adverse effects, human PBMC‐reconstituted NSG mice (*n* = 3) were used as an immune‐humanised model, allowing for the detection of human immune responses caused by the fusion protein (Figure 6A). Throughout the treatment course, no significant differences were observed between the saline control group and hPD1‐CD137L‐treated group in terms of body weight or overall survival (Figure S5A,B). Potential systemic inflammatory responses to hPD1‐CD137L were evaluated by measuring key pro‐inflammatory cytokines. No significant differences were observed in IFN‐γ concentrations in either serum or liver, nor in TNF levels within liver homogenates between saline‐treated and hPD1‐CD137L‐treated mice (Figure 6B,C). TNF was undetectable in the serum of all mice, further indicating the absence of systemic inflammation. These results indicate that repeated administration of hPD1‐CD137L does not trigger measurable immune‐related toxicity in PBMC‐reconstituted NSG mice, thus providing a basis for testing of the hPD1‐CD137L fusion protein in vivo.

**FIGURE 6:**
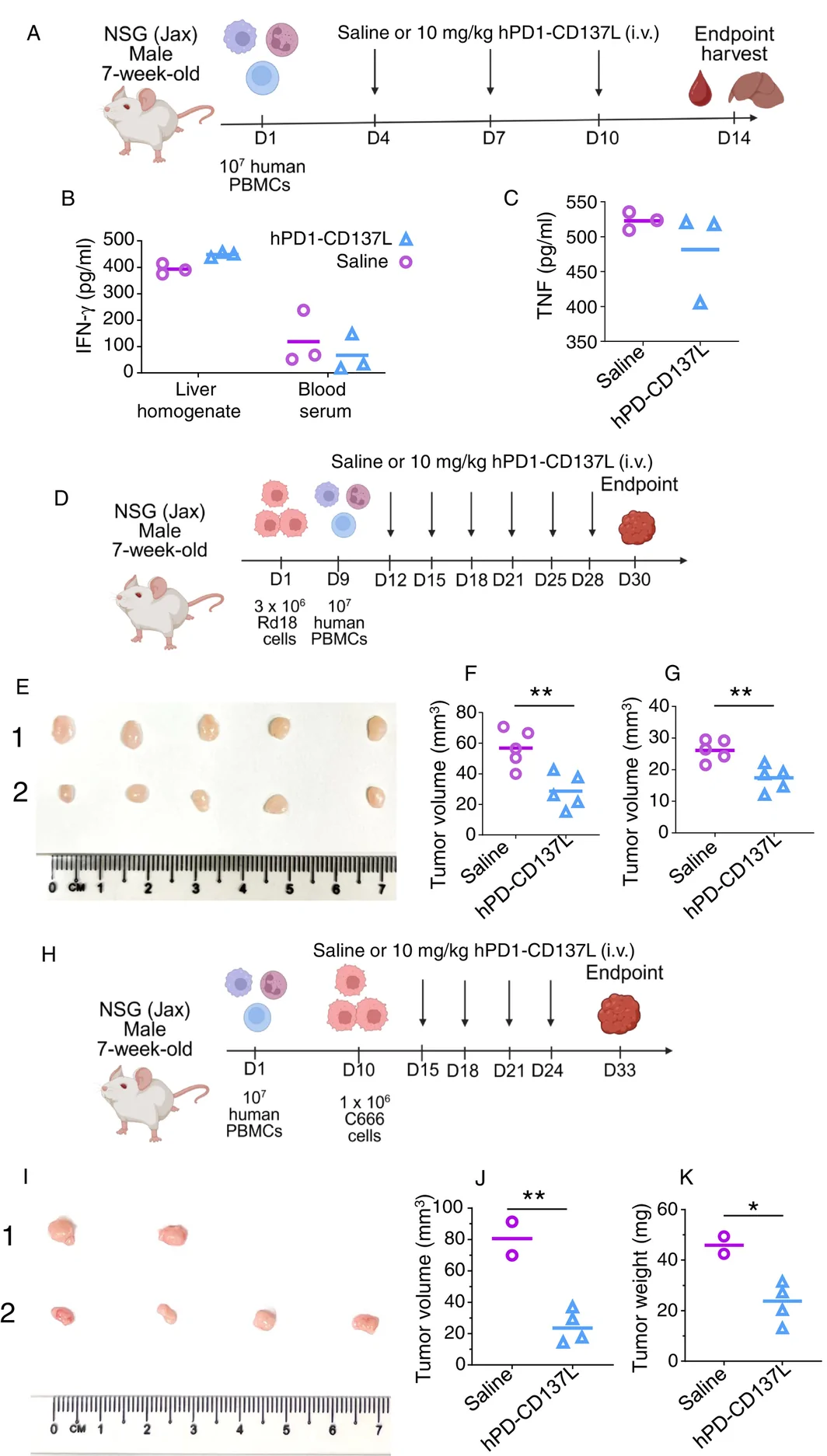
hPD1‐CD137L exhibits a favourable safety and antitumor efficacy profile in PBMC‐reconstituted murine models. (A) Experiment timeline of in vivo toxicity test (*n* = 3). (B) IFN‐γ in blood serum and liver homogenate. (C) TNF profile in liver homogenate. (D) Timeline of in vivo efficacy study in Rd18‐engrafted, PBMCs‐reconstituted humanised mouse model (*n* = 5). (E) Images of harvested Rd18 tumours from both groups where the first row represents the saline‐treated group and the second row represents the group treated with 10 mg/kg hPD1‐CD137L. (F, G) The volume and weight of the harvested Rd18 tumours from both groups as analysed by unpaired *t*‐test. (H) Timeline of in vivo efficacy study in C666‐engrafted, PBMCs‐reconstituted humanised mouse model. (I) Images of harvested C666 tumours from both groups where the first row represents the saline‐treated group and the second row represents the group treated with 10 mg/kg hPD1‐CD137L. (J, K) The volume and weight of the harvested C666 tumours from both groups as analysed by unpaired *t*‐test.

To investigate the therapeutic efficacy of the hPD1‐CD137L fusion protein in vivo, NSG mice were engrafted with Rd18 cells, a human RMS cell line exhibiting the highest PD‐L1 expression among those tested (Figure S6). After the tumours reached a stable and palpable size, human PBMCs were injected intravenously to reconstitute a humanised immune system. The Rd18‐engrafted, PBMCs‐reconstituted mice were randomised into control and treatment groups (*n* = 5) and administered saline or hPD1‐CD137L, respectively, via tail vein injection (Figure 6D). At the experimental endpoint, subcutaneous Rd18 tumours were excised and examined macroscopically. Tumours from mice treated with hPD1‐CD137L were visibly smaller in size compared to those from the saline‐treated control group (Figure 6E). This qualitative observation was substantiated by quantitative analysis of tumour volume and tumour weight. Both the final tumour volume and the excised tumour weight were significantly reduced in the hPD1‐CD137L group relative to controls (Figure 6F,G). Importantly, these tumour‐suppressive effects were achieved without observable adverse outcomes, as evidenced by stable body weight across the study period (Figure S5C).

The antitumor efficacy of hPD1‐CD137L was further validated using a second tumour model based on C666 NPC cells. In contrast to the Rd18 model, C666 cells were implanted subcutaneously after PBMC reconstitution, as C666 tumours readily engraft without the requirement for Matrigel. Following tumour establishment, mice were randomised into saline or hPD1‐CD137L treatment groups (*n* = 5) and administered four intravenous doses of the respective treatments (Figure 6H). Three mice from the saline group and one mouse from the hPD1‐CD137L group were euthanised after the last dose due to significant weight loss, which is likely due to GvHD that often sets in 3–4 weeks after PBMC injection into NSG mice [18]. The remaining animals (*n* = 2 in the control group and *n* = 4 in the treatment group) were euthanised 9 days after the final dose, and tumours were collected for analysis. Gross examination revealed larger tumours in the saline‐treated group compared with the hPD1‐CD137L‐treated cohort (Figure 6I). Consistently, tumours excised from hPD1‐CD137L‐treated mice exhibited a pronounced reduction in overall mass and volume compared to those from saline‐treated animals (Figure 6J,K). The small animal numbers, which likely resulted from the GvHD, of course caution conclusions from this experiment. While by itself it would not be convincing, hPD1‐CD137L treatment exhibited a similar trend in both tumour models. The more pronounced GvHD observed in the C666 model compared with the Rd18 model may be attributed to donor‐to‐donor variability in PBMC composition and/or the more rapid engraftment and growth kinetics of C666 tumours, which could heighten inflammatory stress, enhance systemic xenoreactivity, and thereby exacerbate xeno‐GvHD.

Collectively, these results demonstrate that hPD1‐CD137L can effectively delay tumour growth in vivo, encouraging its development as a bifunctional immunotherapeutic approach for PD‐L1‐positive tumours.

## 4 Discussion

This study demonstrates the successful production and functional validation of the hPD1‐CD137L fusion protein, confirms its structural resilience and establishes its potent anti‐tumour activity across multiple cancer models in vitro and in a humanised xenograft model in vivo.

Structurally, both the PD1 and CD137L domains can bind to their respective target on live cells. Partially denaturing SDS‐PAGE revealed monomeric and trimeric forms of hPD1‐CD137L that were held together by disulphide bonds and remained stable over time and at low pH as found in the TME. For TNF superfamily members such as CD137L, forming multimers not only enhances potency but also improves stability [19, 20]. Stability under mildly acidic conditions is especially relevant, because solid tumours commonly generate an acidic extracellular environment (pH≈6.5–6.9), which can stress protein therapeutics and modulate their activity [21, 22].

In T cell monocultures, hPD1‐CD137L led to the upregulation of activation markers, increased cytokine secretion and NF‐κB pathway activation. In cocultures with T cells from multiple independent healthy donors, hPD1‐CD137L enhanced cytolysis across diverse tumour cell lines, including NPC (C666), RMS (Rd18) and lung adenocarcinoma (A549). Despite the difference in PD‐L1 expression among the three cell lines, of which Rd18 expresses the highest level of PD‐L1 followed by C666 and A549, hPD1‐CD137L consistently demonstrated much higher efficacy in enhancing tumour cell lysis than only targeting the costimulatory CD137 axis or the immunoinhibitory PD‐L1 axis. The hPD1‐CD137L was more effective than targeting CD137 and PD‐L1 at the same time but by separate drugs, which is commonly done in current immunotherapies, known as combination therapies. Fusion of the CD137 and PD‐L1 targeting moieties assures that a T cell receives both signals at the same time thus ensuring better pharmacokinetics and improved signalling synergy.

Clinically, this could be significant because it suggests that hPD1‐CD137L may be active not only in PD‐L1^high^ tumours, where checkpoint blockade alone tends to be effective, but also in PD‐L1^low^ or heterogeneous tumours, which are often less responsive to monotherapy by PD1/PD‐L1 checkpoint inhibitors [1]. The ability of hPD1‐CD137L to retain efficacy across a PD‐L1 expression spectrum positions it as a promising next‐generation immunotherapeutic drug, capable of addressing a broader patient population than conventional ICIs.

To extend these findings to a clinically relevant context, TILs from NPC patients were cocultured with C666 cells, which demonstrated significantly greater tumour cell lysis following hPD1‐CD137L treatment compared to other conditions. Given that TILs reflect the antigen experience, activation state and suppressive influences of the tumour milieu in vivo [23, 24], these findings provide translationally relevant evidence that hPD1‐CD137L may potentiate the cytotoxic capacity of tumour‐resident T cells within the NPC tumour microenvironment. Moreover, the ability of hPD1‐CD137L to promote tumour cytolysis even at low effector‐to‐target (E:T) ratios also highlights an important aspect of its therapeutic potential, particularly in immune‐desert or immune‐excluded tumours, where conventional ICIs often exhibit limited clinical efficacy due to limited T cell infiltration [25, 26]. By amplifying the activity and persistence of the limited TIL populations, hPD1‐CD137L may help to convert these poorly infiltrated tumours to immunologically responsive ones, thus broadening the spectrum of patients who could benefit from T cell–based immunotherapy.

A significant enhancement of T cell‐mediated killing was also observed in patient‐derived colorectal organoids and in humanised murine tumour models in vivo. This underscores the potential of hPD1‐CD137L physiologically in more complex settings. The in vivo toxicity study of hPD1‐CD137L in PBMC‐reconstituted NSG mice showed no adverse impact on mouse weight or survival, and cytokine profiling revealed no significant increase in serum or liver IFN‐γ or TNF levels compared to the saline‐treated control group. Moreover, preliminary efficacy evaluation in PBMC‐reconstituted NSG mice bearing subcutaneous Rd18 tumours further supports the translational promise of hPD1‐CD137L. Treatment with hPD1‐CD137L showed reduced tumour volume without observable toxicity, as indicated by stable body weight and unaltered survival over the 30‐day study period. These results suggest that hPD1‐CD137L can activate anti‐tumour immune responses without inducing systemic inflammatory toxicity, a key consideration for clinical translation.

This study also has its limitations, among them are a low number of donor cells in several experiments which is partly due to the scarcity of TILs. We tried to compensate for this by demonstrating comparable results using different tumour cell lines as targets. The limited numbers used for in vivo toxicity analysis of hPD1‐CD137L (*n* = 3) and 2‐week assessment do not entirely rule out the adverse effects associated with the protein. However, the absence of systemic inflammation and weight loss provided sufficient confidence to conduct the preliminary in vivo experiments. Finally, the PBMC‐humanised NSG mouse models employed present several limitations, particularly xenogeneic graft‐versus‐host disease (xeno‐GvHD), which is well documented to hinder accurate efficacy evaluations. The use of MHC‐deficient NSG mice will be considered to allow a more in‐depth evaluation of the protein.

Several bispecific antibodies that target PD‐L1 and CD137 have entered preclinical and early clinical development, including MCLA‐145, GEN1046 and IBI319 [7, 8, 9]. However, these bispecific antibodies depend on the presence of both PD‐L1 and FcγR engagement within the TME for effective crosslinking, which may restrict their efficacy in PD‐L1^low^ tumours. Further, since hPD1‐CD137L activates CD137 without Fc‐mediated clustering, it is unlikely to carry the risk of Kupffer cell‐mediated hepatotoxicity [6]. Rather, the clustering by PD1‐CD137L correlates with the levels of PD‐L1, which means the more PD‐L1 a tumour expresses, the more PD1‐CD137L it will bind and the stronger the T cell costimulation will be.

Unlike bispecific antibodies, the hPD1‐CD137L fusion protein consists of the native receptor/ligand domains which more closely mimic physiological ligand–receptor interactions and thus foster the clustering of CD137 receptors on T cells. Together with the delivery of the two signals, this can potentiate stronger costimulatory signalling. In addition, hPD1‐CD137L is of a smaller molecular size, which is expected to improve tissue penetration.

In summary, these findings support hPD1‐CD137L as a candidate for next‐generation cancer immunotherapy, capable of combining immune checkpoint blockade with CD137‐mediated costimulation in a structurally stable and functionally potent format. Its consistent efficacy in various in vitro assays and in vivo cancer models, along with encouraging safety data, argues for continued development and clinical translation.

## Author Contributions

R.X. designed and performed all experiments aside of those indicated otherwise. B.L. performed the competitive binding study. E.P.N. helped in protein production and purification. Y.M. helped with animal experiments. T.M. and G.C. provided tumour spheroids. Y.T.P. and C.M.L. provided NPC patient‐derived TILs. R.S. and K.Y.L. participated in data interpretation and discussion. R.X. and H.S. wrote the paper, and all authors revised it. H.S. supervised the research.

## Funding

This work was supported by National Research Foundation Singapore, CRP28‐2022RS 0001. National Medical Research Council, Singapore, OFIRG25jan‐001.

## Conflicts of Interest

The authors declare no conflicts of interest.

## Supporting information


**Figure S1:** hPD1‐CD137L predominantly exists in trimeric and monomeric forms, which remain stable over time and at different pH. (A) Multimeric status of the fusion protein across three batches of production. (B) Ratio of trimeric and monomeric forms of the protein in western blot as quantified by ImageJ and statistically analysed by paired *t*‐test in Prism. (C) Disulphide bond–mediated stabilisation of protein multimers. (D, E) Binding integrity of PD1 domain or CD137L domain to Rd18 cells or HEK293‐CD137 cells respectively for fusion proteins stored for different durations at 4°C. (F, H) Binding integrity of PD1 domain to Rd18 cells cultured in RPMI at different pH induced by varying concentrations of lactic acid (LA). (I–K) Binding integrity of CD137L domain to HEK293‐CD137 cells cultured in DMEM at different pH induced by varying concentrations of lactic acid (LA). (L) Multimeric status of the fusion protein at different pH.
**Figure S6:** Flow cytometry scatter plot of % human PD‐L1 expression in (A) Rd18, (B) C666 and (C) A549 with respect to isotype control.

**Figure S2:** Competitive binding of the PD1‐CD137L fusion protein and Atezolizumab. Flow cytometry histograms showing the binding of the PD1‐CD137L fusion protein to Rd18 cells. Prior to staining, the Fc domain was blocked to prevent non‐specific antibody staining mediated by Fc regions. The fusion protein was subsequently detected using antibodies against the CD137L (A) and the PD‐1 (B) domains. Values represent the median fluorescence intensity (MFI).
**Figure S3:** Immunophenotype of ex vivo expanded tumour‐infiltrating lymphocytes (TILs) from treatment‐naive nasopharyngeal carcinoma (NPC) tissue biopsies. Expanded TILs from dissociated NPC tissue from 2 patients were immunophenotyped after 2–3 weeks culture with recombinant IL‐2 (Proleukin). Shown are percentages of CD3+ T cell and CD56+ NK cell population gated on viable lymphocytes, followed by percentages of CD8+ cytotoxic T lymphocytes (CTL) and CD4+ T cells gated on CD3+CD56− T cells.
**Figure S4:** Individual tumour cytolysis by T cells from 5 independent healthy donors.
**Figure S5:** (A) Survival and average weight of mice over time in toxicity study. (B) Average weight and tumour volume over time of efficacy study. Statistical analysis was done via Two‐way ANOVA in Prism.

## Data Availability

All data that led to the conclusions in this manuscript have been referenced and all sources have been described. The data that support the findings of this study are available from the corresponding author, H.S., upon reasonable request.
